# Iron Load Toxicity in Medicine: From Molecular and Cellular Aspects to Clinical Implications

**DOI:** 10.3390/ijms241612928

**Published:** 2023-08-18

**Authors:** George J. Kontoghiorghes

**Affiliations:** Postgraduate Research Institute of Science, Technology, Environment and Medicine, 3, Ammochostou Street, Limassol 3021, Cyprus; kontoghiorghes.g.j@pri.ac.cy; Tel.: +357-26272076

**Keywords:** thalassemia, hemochromatosis, carcinogenesis, iron overload, iron toxicity, free radicals, Fenton reaction, ferroptosis, mechanisms, diagnostic techniques

## Abstract

Iron is essential for all organisms and cells. Diseases of iron imbalance affect billions of patients, including those with iron overload and other forms of iron toxicity. Excess iron load is an adverse prognostic factor for all diseases and can cause serious organ damage and fatalities following chronic red blood cell transfusions in patients of many conditions, including hemoglobinopathies, myelodyspasia, and hematopoietic stem cell transplantation. Similar toxicity of excess body iron load but at a slower rate of disease progression is found in idiopathic haemochromatosis patients. Excess iron deposition in different regions of the brain with suspected toxicity has been identified by MRI T2* and similar methods in many neurodegenerative diseases, including Alzheimer’s disease and Parkinson’s disease. Based on its role as the major biological catalyst of free radical reactions and the Fenton reaction, iron has also been implicated in all diseases associated with free radical pathology and tissue damage. Furthermore, the recent discovery of ferroptosis, which is a cell death program based on free radical generation by iron and cell membrane lipid oxidation, sparked thousands of investigations and the association of iron with cardiac, kidney, liver, and many other diseases, including cancer and infections. The toxicity implications of iron in a labile, non-protein bound form and its complexes with dietary molecules such as vitamin C and drugs such as doxorubicin and other xenobiotic molecules in relation to carcinogenesis and other forms of toxicity are also discussed. In each case and form of iron toxicity, the mechanistic insights, diagnostic criteria, and molecular interactions are essential for the design of new and effective therapeutic interventions and of future targeted therapeutic strategies. In particular, this approach has been successful for the treatment of most iron loading conditions and especially for the transition of thalassemia from a fatal to a chronic disease due to new therapeutic protocols resulting in the complete elimination of iron overload and of iron toxicity.

## 1. Introduction

Iron is an essential metal found in all the cells of the body, playing an important role in many physiological and cellular processes. There are also many diseases associated with iron metabolism affecting many people. In particular, diseases of iron imbalance affect billions of patients, mostly with iron deficiency anemia. There are also many diseases associated with iron overload and other toxicity. In this context, the presence of excess iron is an adverse prognostic factor for all diseases. Especially in heavily iron loaded patients, iron can cause serious organ damage and fatalities.

Millions of patients with hemoglobinopathies and other refractory anemias worldwide, including thalassemia, sickle cell disease (SCD), myelodyspasia, and hematopoietic stem cell transplantation (HCT), receive regular red blood cell (RBC) transfusions for the treatment of their anemia [1,2,3,4,5]. 

A major side effect of chronic RBC transfusions is the build-up of excess, toxic deposits of iron in various organs, which results in tissue damage and organ malfunction [6,7,8]. Congestive cardiac failure is the main cause of mortality in heavily iron-loaded beta thalassemia major (TM) patients who have no access to chelation therapy for the removal of excess iron [9].

A gradual increase in body iron levels from chronic increases in dietary iron absorption and the cause of iron overload toxicity in the liver and other organs was also observed in idiopathic haemochromatosis, which is a genetic disease affecting one in ten people of the Caucasian population [10]. 

The assessment of excess iron levels in different categories of iron-loaded patients is determined mainly from the estimation of iron diagnostic parameters such as serum ferritin, iron concentration from liver biopsies, and magnetic resonance imaging (MRI T2*) measurements. Iron toxicity may affect the function of many other organs in addition to those observed in iron-loaded patients. For example, iron accumulation in parts of the brain was detected using MRI T2* in many neurodegenerative and other diseases [11]. 

The presence of excess iron deposition or labile forms of iron in any part of the body is a potential hazard and source of continuous toxicity [6,9]. In most cases, iron overload toxicity in moderate iron-loaded organs is reversible and the restoration of normal function can be accomplished by the removal or reduction of excess iron deposition. The sooner the removal of excess or labile forms of iron can be accomplished, the lower the level of permanent damage in the organs affected [12].

The rate of body iron intake from chronic RBC transfusions is in most cases the major assessment parameter for the selection of effective chelating drug protocols for achieving negative iron balance in chronically transfused patients. In this context, the efficacy in the removal of excess iron by chelating drugs requires their daily use for such categories of iron-loaded patients. Iron chelation therapy worldwide is carried out using deferoxamine (DF), deferiprone (L1) and deferasirox (DFRA) [7,13]. The combination of chelating drugs, and especially the combination of L1 and DF, is also widely used in TM patients in many countries [14,15,16]. 

The main aim of iron chelation therapy in TM and other chronically transfused patients is the design of effective chelating drug protocols for the achievement of negative iron balance, which can lead to the reduction of the excess iron load. The ultimate aim of chelation therapy is the achievement and maintenance of normal iron stores, in which case patients are devoid of all iron overload toxic side effects and consequently the overall reduction in morbidity and mortality in transfusional iron-loaded patients [17]. This aim, including the long-term prevention of iron overload, can be accomplished in most cases using effective and safe chelation protocols [17]. Furthermore, regular monitoring of the iron load and selected chelating drug dose protocols will also be needed to ensure that no prolonged iron deficiency or chelating drug toxicity can be caused in TM and other categories of chronically transfused patients that have reached the stage of normal iron store levels.

The introduction of effective therapies and prophylactic measures is also necessary in all other diseases of iron overload and or toxicity. In each case the causes, affected sites, and levels of iron toxicity have to be identified, analyzed, and monitored for introducing effective protocols, which can achieve complete therapy of the disease or optimal therapeutic outcomes. Such approaches will require the understanding of the mechanisms of toxicity from the molecular level to the whole organism. In this context, the underlying mechanisms of iron toxicity in primarily transfusional iron overload will mainly be discussed, since this is considered the most affected disease of metal intoxication with the highest morbidity and mortality rate worldwide.

Other forms of iron toxicity will also be discussed for many other diseases, including ferroptotic cell death, which is identified as one of the most common but also rare human diseases, the presence of focal iron deposits in non-iron-loaded conditions, especially in neurodegenerative diseases, and also of iron diversion in the reticuloendothelial system in the anemia of chronic disease categories. Further discussion includes different forms of labile iron toxicity in other clinical conditions associated with tissue damage and free radical pathology, toxic forms of carcinogenic iron complexes in food and environmental pollution, and also other forms of toxicity arising from molecular interactions of iron with dietary, biochemical, drug, and other xenobiotic molecules.

## 2. The Source of Iron Overload from Chronic Red Blood Cell Transfusions

The main source of iron overload in regularly RBC transfused patients with refractory anemia is the blood obtained from blood donors. The quantitative aspects related to body iron intake from RBC transfusions and the metabolic pathways of iron accumulation in different organs provide important information for assessing therapeutic strategies. In particular, the information on the properties and the role of RBC, as well as its components, is crucial for understanding the extent of the complications of iron overload.

**Figure 1:**
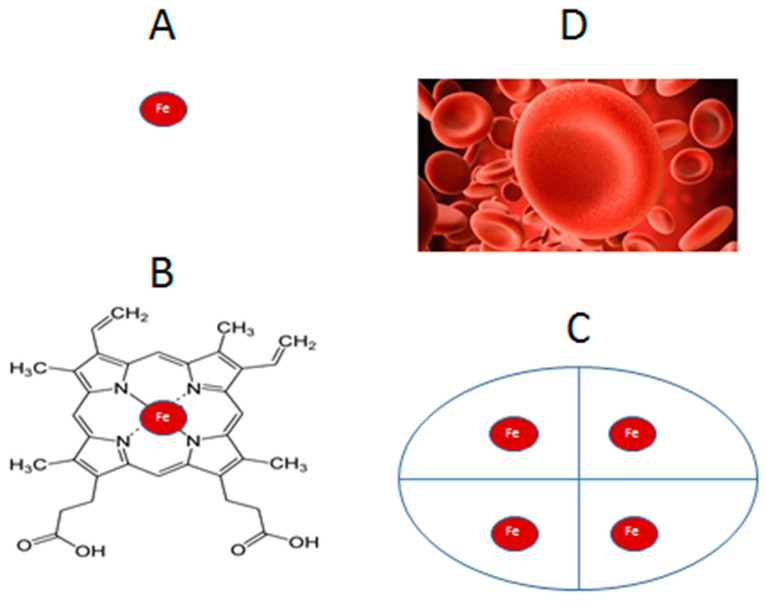
The important role of iron in heme, hemoglobin, and red blood cells. A cartoon representation of the essential role of iron (red circle, (**A**)), which is responsible for the red color of blood. Iron is embedded in a protoporphyrin ring forming heme (**B**), which is the prosthetic group in each of two alpha and two beta globin chain components of the protein hemoglobin (**C**). Each hemoglobin molecule (**C**) contains four molecules of iron, which are responsible for binding and carrying oxygen. The protein hemoglobin is the main constituent (95%) of red blood cells, as shown in the picture in (**D**).

In considering the qualitative and quantitative aspects of iron distribution in man, it is estimated that about 58% of the iron in the body is found in the form of heme in hemoglobin, which is the major constituent of RBC and the component that carries oxygen and gives the red color to blood (Figure 1). The role and function of RBC and hemoglobin are crucial in respiration and other functions. In particular, normal cellular, tissue, and bodily functions can only be ensured if a sufficient and continuous supply of oxygen is available to all parts of the body through blood circulation and also if sufficient normal RBC and hemoglobin levels are available to carry over the necessary amounts of oxygen.

The quantitative aspects in relation to oxygen transport by RBC and hemoglobin and the role of iron in heme, where oxygen is bound, is of primary importance for normal bodily functions and overall human survival. Hemoglobin is estimated to occupy 95% of the RBC volume and amounts to about 670 g of the 25 kg dry body weight (2.7%) of an average human adult individual [18,19,20]. Furthermore, it is estimated that approximately 25 trillion RBC circulate in the bloodstream, each one packed with about 260 million hemoglobin molecules. Considering that one adult hemoglobin molecule is composed of two alpha and two beta globin protein subunits, to each of which one molecule of heme is embedded, the total amount of iron in the ferrous state as heme in one RBC is estimated to be 1.04 billion iron molecules (Figure 1). In this context, each unit of RBC transfused contains about 200–250 mg of iron, which is added to the body iron burden.

There are many other unique characteristics of RBC in relation to heme iron, such as the high concentration of both iron and oxygen, which is estimated to reach about 16 mM when the RBC are fully oxygenated. It should be noted that no other cell contains such a high concentration of protein, in this case hemoglobin with iron in the ferrous state in heme and also oxygen. This high concentration of ferrous iron and oxygen can potentially be a highly reactive mixture. This can lead to reactive oxygen species and other free radicals resulting in oxidative stress toxicity, especially in hemolytic and other damaging effects involving RBC [19,20,21,22]. In most cases, strict metabolic controls and the presence of antioxidant enzymes such as superoxide dismutase and molecules such as glutathione ensures the antioxidant protection inside the RBC [23].

There are different stages in the production and development of RBC and their release in the blood circulation, where on average they have a life span of 120 days. Erythroblasts are the early-stage RBC progenitor cells containing a nucleus and produced in the bone marrow, which progressively lose their nucleus and organelles, before their release in the bloodstream initially as reticulocytes and later as matured RBC. Heme production takes place in mitochondria and the protein part of hemoglobin is produced in the cytoplasm of both erythroblasts and reticulocytes. In contrast, mature RBC cannot synthesize new proteins during their 120 day lifespan in the human bloodstream [19,20,24].

At the end of their lifespan aging RBC undergo progressive structural and denaturing changes such as vesiculation, a process leading to the formation of vesicles [24,25,26]. Furthermore, senescent RBC become more rigid and fragile than younger RBC, with the former being identified and readily removed from the bloodstream via phagocytosis by macrophages of the reticuloendothelial system primarily in the spleen and the liver. The degradation process begins inside the macrophages and usually involves only old and damaged RBC, where iron is released from heme and stored initially in the reticuloendothelial cells and then released in the bloodstream, taken by plasma iron transporter protein transferrin and distributed in many organs for storage and re-utilization [20,24,25,26].

In addition to the main source of iron overload, which is iron from senescent RBC, many other sources contributing to iron body burden and associated iron toxicity can also take place in hemolytic and other events, which are observed in many of the categories of patients with refractory anemia receiving RBC transfusions. In this context and under normal physiological conditions, several innate mechanisms are in operation for the control of iron metabolic pathways and protection against iron toxicity. For example, the removal of denatured hemoglobin or other aggregated species containing iron inside the RBC is accomplished by a vesiculation process [23,24,25,26]. Similarly, protection against oxidative and other damage caused by the release of heme and hemoglobin into the blood stream during hemolysis is undertaken by hemopexin and haptoglobin, respectively [27,28]. Hemopexin is a plasma protein expressed mainly in the liver and has a high affinity to heme binding. Similarly, haptoglobin in plasma binds free hemoglobin released from RBC forming a haptoglobin–hemoglobin complex, which is removed by the reticuloendothelial system in the spleen, where, after a degradation process, iron and other components are released to follow their corresponding metabolic pathways [27,28].

Several toxicity and various other factors leading to changes in the concentration levels of iron, oxygen, hemoglobin, and RBC can also cause many other abnormalities and side effects, including anemia and insufficient transport of oxygen to the tissues. In such cases, the general function of all the cells, tissues, and organs of the body is undermined, leading to reversible or irreversible damage and the need for therapeutic intervention.

## 3. Major Diseases of Transfusional Iron Overload

Millions of hematologic and other categories of patients worldwide suffer from different types of anemia, in addition to iron deficiency anemia. Among these, there are many conditions with abnormalities in the structure and function of RBC and hemoglobin, where RBC transfusions may be required to maintain normal bodily functions (Table 1). For example, hundreds of hemoglobin mutations have been reported in humans, where in many cases the changes in the production rate and structural characteristics of normal hemoglobin can lead to abnormal function, toxic side effects, and anemia [29].

The hemoglobinopathies are the most common group of genetic disorders affecting millions of people worldwide, mostly in the developing countries [29,30,31]. In this context, there are many abnormalities in the function of hemoglobin which are related to changes in the globin structure and heme function (Figure 1). For example, patients with thalassemia have a low or absent production of the alpha or beta or other globin chains of hemoglobin (Table 1). Most TM patients cannot produce the beta globin chain of hemoglobin and are therefore severely anemic, requiring RBC transfusions from normal blood donors in order to survive [1,29,30]. Another inherited hemoglobinopathy affecting millions of people is SCD, which is prevalent in Africa and many other countries with descendants from Africa. In this abnormal hemoglobin condition, a single amino acid change in the beta globin chain can cause hemoglobin polymerization, sickling of RBC, anemia, and painful side effects as a result of the sickling crisis [3,29,30]. Treatment with RBC transfusions improves anemia and alleviates some categories of the SCD patients from the painful side effects of the sickling crisis.

In the absence of effective excretory iron mechanism in humans, the build-up of body iron burden progressively increases with each RBC transfusion. Considering that each unit of transfused RBC contains about 200–250 mg of iron and that a total 4.5–5.0 g of iron is present in the body of a 70–75 kg normal adult man, it is estimated that for every 25 RBC units transfused, the total body iron burden is doubled [20]. Under these conditions, different amounts of excess iron accumulate progressively in the body, which depends on the rate of RBC transfusions, considering that there are different requirements in each patient category and for each individual patient (Table 1).

Contribution to body iron burden from increased absorption of dietary iron is observed in TM and other categories of regularly RBC transfused patients, especially at periods of low concentration of hemoglobin and increased anemia prior to transfusion. This process involves a body intake of much smaller amounts of less than 6 mg of iron per day, in comparison to that obtained from transfusions [32,33,34,35]. In some cases, such as thalassemia intermedia (TI) or non-transfusion dependent thalassemia and similar conditions, the frequency of transfusions, if any, is significantly reduced (Table 1) [34,36,37,38]. In this context, there is a large difference between the genotypes in thalassemia and RBC transfusion requirements to the extent that increased iron absorption in most cases of TI is the dominant route of body iron intake and loading in comparison to that of iron intake from transfusions [34,36,37,38].

Many other differences in hematologic disease characteristics and therapeutic requirements, including the initiation and rate of RBC transfusions, exist between the various categories of transfusional iron-loaded patients (Table 1) [1,2,3,4]. For example, the initiation of RBC transfusions ranges from the first few years to the rest of remaining life in TM, whereas in SCD and TI they may be intermittent at different patient ages [3,36,37]. In contrast, in the myelodysplastic syndromes the patients affected are mostly of middle to old age and transfusions are usually initiated later in life [4]. Similarly, the rate of RBC transfusions may also vary, with transfusion frequency every 1–4 weeks in TM and a much lower rate for many TI patient categories. Further variations on RBC transfusions and rate of iron loading may be related to the underlying disease, individual and other factors such as hypersplenism, hemolysis, and RBC antibodies [39].

Limitations regarding RBC transfusion management may also be caused by factors related to changes in the underlying disease and as a result of variable effects due to the poly-pharmacotherapy in each case. For example, HCT is a widely used form of treatment for many hematologic malignancies and genetic disorders. The number of diseases and the number/age range of patients undergoing HCT is rapidly increasing as a result of technological and therapeutic management improvements (Table 1) [2,40,41,42,43].

The level of iron overload caused from RBC transfusions is considered an independent adverse prognostic factor in all diseases associated with HCT and is one of the many complications in pre- and post-transplantation side effects, affecting the long-term survival of about 0.5 million HCT patients [2,40,41,42,43]. Iron overload mostly develops in the pre-transplantation period in many HCT patient categories, including about 4000 patients with TM and 1000 with SCD, as well as many more with myelodysplasia, leukemia, and other patients [2,40,41,42,43]. The excess iron burden in the transfusion-independent or reduced transfusion dependent post-allogeneic iron-loaded HCT patients always remains a potential source of toxicity and clinical complications unless it is removed.

In addition to the different rate of RBC transfusions between the various hematologic and other categories of iron-loaded patients, there are also many patient categories needing transfusions because of different causes of anemia, such as sideroblastic anemia, where iron is deposited in the mitochondria of sideroblasts [44,45,46]. In all RBC, transfusional iron-loaded categories and non-transfusional iron-loaded categories such as idiopathic hemochromatosis patients where venesection is contraindicated, iron chelation therapy needs to be introduced in order to reduce or eliminate the iron toxicity (Table 1) [34,47,48].

The major category of hematologic diseases in need for lifelong RBC transfusions with the highest number of patients and daily iron chelation therapy is TM, which has the highest number of metal-related mortality and morbidity rate worldwide as a result of iron overload toxicity [1,13,29,30,31]. Thalassemia major is found mainly in developing countries and in the Mediterranean, Middle East, and South-East Asia, where more than 90% of TM patients are born [1,29,30]. It is estimated that worldwide there are 100 million thalassemia heterozygote asymptomatic carriers, and more than 100,000 TM babies are born each year [29,30]. In India, for example, the annual birth rate of TM patients is estimated at 9000 [49].

## 4. Iron Metabolism and Iron Toxicity in Chronically Transfused Patients

Iron is essential for many biological processes, including respiration, hemopoiesis, growth, and development [20,50,51,52,53,54]. The build-up of excess iron in several organs of patients receiving regular RBC transfusions is due to the lack of efficient excretion mechanism of accumulated iron. This is in contrast to the presence of efficient physiologic mechanisms of dietary iron body intake and conservation [20,35,50,51,52,53,54].

Under normal physiologic conditions, the transport, storage, and utilization of iron is controlled by metabolic pathways involving proteins such as transferrin in plasma, transferrin receptors on the cell membrane for intracellular iron uptake through endocytosis, and the presence of intracellular ferritin, which can accommodate up to 4500 iron molecules and is used for iron storage [20,55,56]. Hemosiderin is a breakdown product of ferritin, which is the major intracellular iron storage protein found in iron-loaded tissues of patients with iron overload [6,20,57].

Iron toxicity has been implicated in almost all diseases of tissue damage, including those associated with the presence of redox active iron and iron overload in hematologic and other diseases [20,21,22,54,57,58,59,60,61,62,63,64,65]. The basic molecular mechanism of iron toxicity is related to the role or association of iron as the main biological catalyst in free radical production and related pathology [20,57,58,59,60,61,62,63,64,65].

The general mechanism of free radical production primarily includes the Fenton reaction, which involves Fe(II) and hydrogen peroxide and leads to the catalytic formation of the highly reactive and toxic hydroxyl radicals with a half-life of reactivity within micro- to nano-seconds. Similarly, the production of other free radicals such as superoxide and nitrous oxide, as well as oxygen-activated products such as hydrogen peroxide, can all be involved in oxidative chain reactions and cascades, leading to oxidative damage to virtually all known organic biomolecules, including DNA, proteins, sugars, and lipids [54,58,59,60,66,67]. Several other toxicity mechanisms involving different forms of iron ions such as Fe(IV) and reducing agents such as ascorbate and chelating molecules may also enhance free radical toxicity [54,58,59,60,63,64,66,67].

**Figure 2:**
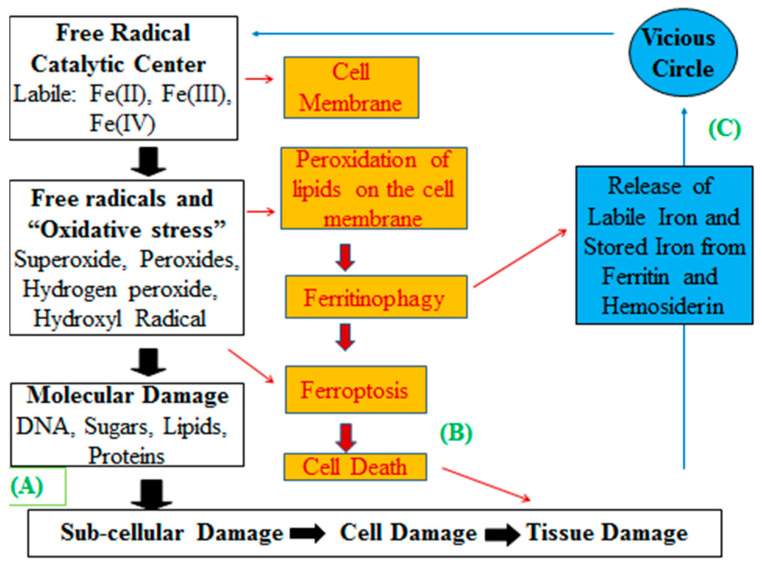
The catalytic role of iron in free radical pathology and ferroptosis. Iron catalyzes the formation of free radicals, mainly the hydroxyl radical (OH), through the Fenton reaction, causing oxidative stress and leading to molecular, subcellular, cellular, and tissue damage (**A**). A similar pathway is followed in ferroptosis, where iron mostly from ferritinophagy catalyzes the formation of free radical cascades, causing lipid peroxidation on the cell membrane and cell death (**B**). Cell and tissue damage and ferroptotic cell death cause the release of more iron, resulting in a vicious circle of free radical production (**C**).

Within this context, the presence of excess iron, mainly in the form of intracellular hemosiderin and associated low molecular weight (LMWt) labile iron, can progressively cause molecular, cellular, and tissue damage, and a vicious circle leading to oxidative stress toxicity damage in cells (Figure 2) [57,65,66,67].

The iron-induced oxidative stress toxicity damage can cause abnormalities to cell structure and function, including damage to cell membrane mainly through lipid peroxidation and organelle damage, such as the disruption of lysosomes causing the release of proteolytic enzymes and resulting in the malfunction of the cellular processes, all of which can lead to cell death such as ferroptosis, apoptosis, autophagy, and necrosis [54,60,66,67].

Under normal conditions, cell death is an essential process for controlled development and the maintenance of homeostasis, as well as for the prevention of proliferative diseases. The importance of iron in cellular function processes including cell death has been recently highlighted by the discovery of ferroptosis, which is a newly described programmed cell death based on oxytosis, which is caused by free radicals arising from iron catalysis and involving transcription and many other factors [68,69,70]. Ferroptosis has been implicated as an important process not only in health but also in diseases, including cancer, COVID-19, hematologic, neurodegenerative, cardiac, kidney, infectious diseases, and many others [71,72,73,74,75,76,77,78,79,80]. Ferroptosis is morphologically, biochemically, and genetically distinct from apoptotic and necrotic cell death and is mainly characterized by the accumulation of lipid peroxides on cell membranes, decrease in glutathione production, inhibition of glutathione peroxidase (GPX4) activity, an increase in intracellular iron, the sustainable production of increased free radical reactions caused mainly by labile but also other forms of iron, ferritinophagy, and an iron-dependent autophagic cell death program (Figure 2) [68,69,70,81,82,83,84,85].

**Figure 3:**
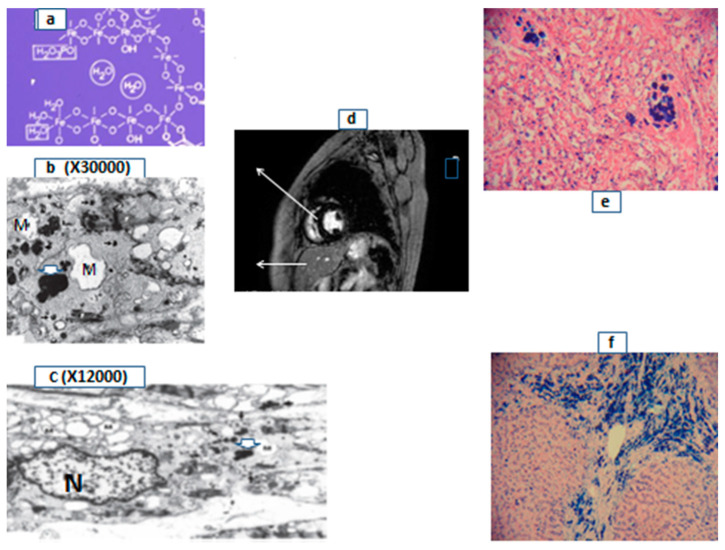
Iron overload toxicity from molecular aspects to effects in organs. (**a**) A cartoon showing oxyhydroxide polynuclear iron complexes resembling the molecular composition of the iron core in the iron storage proteins ferritin and hemosiderin. This is the form of excess iron detected in iron-loaded cells, tissues, and organs of iron-loaded patients. Some water and phosphate molecules are also shown to be associated with the iron core in ferritin. (**b**) Electron micrograph at a ×30,000 magnification with subcellular details of a part of a myocyte, obtained from a cardiac biopsy of an iron-loaded thalassemia patient, who suffered a congestive cardiac failure. It shows extensive iron deposition in the cytoplasm mostly in primary (black arrows) and secondary (white arrowheads) lysosomes, loss/damaged myofilaments (F) and swollen/damaged mitochondria (M). (**c**) Another electron micrograph at a ×12,000 magnification with similar details as (**b**) and an electron density of myocyte nucleus (N) with increased amounts of heterochromatin. (**d**) Magnetic resonance imaging picture of an iron-loaded thalassemia patient, showing differential iron loading of the heart and liver. The top arrow shows the heavy iron deposition in the interventricular septum of the heart of MRI T2* = 6.32 ms (normal T2* = >19), with low signal intensity (dark). The bottom arrow shows the liver of the patient with no excess iron deposition (MRI T2* = 19.2 ms, normal). (**e**) A spleen section of an iron-loaded thalassemia patient, where iron deposits were stained with Pearl’s Prussian blue. There are hemosiderin deposits within cytoplasm and nucleus of macrophages (grade 3) in blue color. (**f**) A liver section of an iron-loaded thalassemia patient, where iron deposits are stained with Pearl’s Prussian blue. There are hemosiderin deposits in hepatocytes and Kupffer cells and within bile ducts (grade 4) in blue color (Adapted with permission from References [88,104]).

Further information on iron overload toxicity in TM at the cellular and ultrastructural level has been obtained from electron microscopy studies [6,57]. In such cases, more detailed information was received from biopsy samples obtained from iron-loaded TM patients containing cardiomyocytes and hepatocytes, where the presence of iron-loaded ferritin arrays, mainly in primary lysosomes and hemosiderin iron aggregates in secondary lysosomes, was observed [6,57,85,86,87,88,89]. Ultrastructural observations of cardiomyocytes have also shown the presence of iron-laden lysosomes, which rupture into the cell sap, causing intracellular damage including large cytoplasmic vacuoles, swollen mitochondria with no iron deposits but with loss of their cristae, substantial loss of myofilaments, an increase in the electron density of nuclei, and also increased amounts of heterochromatin (Figure 3b,c) [86].

Iron overload toxicity in iron-loaded patients can cause damaging effects in many organs, including malfunction of the heart, liver, spleen, and pancreas, and also different complications including congestive cardiac failure, liver fibrosis and cirrhosis, diabetes mellitus, and diminished growth and infertility in teenage TM patients [5,6]. Organ damage in iron-overloaded conditions is progressively developed and can reach different stages, which in general depends on the level of iron load. Early stages of damage in patients’ organs can be detectable when approximately 20–50 units of RBC have been transfused [9,29,90,91,92,93,94,95,96]. At these early stages the organ damage can be reversible provided effective iron chelation therapy protocols are applied. However, in the absence of effective chelation therapy and following repeated RBC transfusions, the organ damage caused by increasing iron load can progressively become irreversible, e.g., in cardiac failure and liver fibrosis.

The major cause of mortality in regularly transfused iron-loaded TM patients not receiving chelation therapy is iron overload toxicity-related cardiomyopathy (Figure 3d) [9]. In this context, MRI diagnostic studies of iron load deposition in the heart of TM patients have shown the existence of a correlation between cardiac damage and the level of cardiac iron load. In particular, there is an increased risk of congestive cardiac failure in TM patients with excess cardiac iron deposition levels [97,98,99,100].

Iron overload can also promote microbial infections, which is the second major cause of morbidity and mortality observed in iron-loaded TM patients [101,102,103]. Similar adverse effects from microbial infections can also be seen in regularly RBC transfused immuno-compromised iron-loaded patients, and especially in HCT patients.

## 5. Diagnosis and Monitoring of Transfusional Iron Overload

The level of excess iron in the body of iron-loaded patients can be diagnosed and monitored using a number of diagnostic techniques, most of which are also generally used for the monitoring of other diseases of iron metabolic imbalance. In addition to monitoring iron level parameters, regular clinical and biochemical assessment is also widely used in order to evaluate organ function and the extent of damage caused by iron overload, as well as other complications of the underlying disease [1,39,89].

The general routinely-used method for estimating body iron stores is blood sample estimation of serum ferritin, serum iron, and transferrin iron saturation. The physiologic non-toxic levels of iron are usually characterized by the normal range of serum ferritin values (male 40–340 μg/L and female 14–150 μg/L), transferrin saturation (25–35%), and serum iron (10–40 μmol/L). In rare cases, urinary iron excretion in response to chelating drugs such as DF and L1 may also be used [39,89].

Another diagnostic method widely used for the estimation of the iron load in regularly transfused and other patients is the measurement of iron concentration in liver biopsy samples, which usually reflects, to some extent, the level of total body iron stores but not the distribution of excess iron (Figure 3f). Liver iron concentration levels, equivalent to not more than 2 mg iron per g liver dry weight, are considered as the maximum normal physiologic levels in the assessment of liver biopsies, whereas higher levels of iron concentration have been classified to various grades of liver iron load, with the higher grades being associated with liver cirrhosis and fibrosis [1,7,9,104,105]. Furthermore, the use of histochemical techniques of the liver biopsy samples can lead to the identification of the non-heme iron concentration and the level, as well as the pattern of iron deposition in hepatocytes and Kupffer cells [39,89,104,105]. However, a drawback of the iron load estimation by this method is that the distribution and concentration of iron in liver and spleen biopsy samples is not uniform (Figure 3e,f) [104]. Another drawback of this method, which was identified by MRI T2* signal intensity relaxation studies, is that liver iron levels and serum ferritin levels do not reflect the iron load or distribution in other organs, such as the heart [106,107,108,109].

In the last 30 years, the newly developed non-invasive techniques of iron estimation using the MRI T2 and T2* signal intensity relaxation times have become the most useful tools for assessing differential organ iron deposition and, in particular, cardiac iron load levels, which are considered the most critical tool in the prognosis of transfused iron-loaded patients [97,98,99,100,104,106,107,108,109]. The concentration of excess iron in the heart was, until recently, impossible to identify by using biopsies or other methods. Furthermore, the introduction of the MRI T2 and T2* techniques have successfully been used for estimating the level of iron loading not only in the heart but also in the liver, spleen, pancreas, and other organs (Figure 3d) [39,96,100,104,108,109,110].

There are different ranges and classifications of the level of iron overload using MRI in relation to signal intensity relaxation times in different organs. In particular, patients with cardiac MRI T2* relaxation times lower than about 8 ms, are considered to be in the heavy hemosiderosis range and to be in danger of cardiac failure. Similarly, moderate cardiac hemosiderosis is considered for patients with cardiac MRI T2* relaxation times in the range of about 8–12 ms, mild hemosiderosis in the range of about 12–20 ms, and above 20 ms to be in the normal range of cardiac iron levels (Figure 3d) [39,100,104,108,109,110].

Different classifications have been described for liver iron overload where MRI T2* relaxation times have been compared to the iron content of liver biopsy samples. Accordingly, MRI T2* signal intensity relaxation times lower than 1.4 ms are estimated to contain more than 10 mg of iron per g dry weight of the biopsy sample and considered to be in the severe hepatic hemosiderosis range. Moderate hepatic hemosiderosis is considered for MRI T2* signal intensity relaxation times in the range 1.4–2.7 ms, corresponding to 5–10 mg/g dry weight, mild in the range of 2.7–6.3 ms corresponding to 2–5 mg/g dry weight, and normal hepatic iron for higher than 6.3 ms corresponding to less than 2 mg/g dry weight (Figure 3d) [39,104,108,109,110]. Although most of the MRI T2 and T2* signal intensity relaxation time techniques were used for the monitoring of iron load in TM, the same methods could also be applied for all the different categories of iron-loaded patients, including SCD, HCT, and myelodysplasia [110,111,112].

The introduction of the MRI T2 and T2* signal intensity relaxation time techniques in combination with histochemistry of mainly liver biopsy samples and electron microscopy have contributed to the evaluation of the mechanisms of iron deposition and toxicity in various organs of iron-loaded patients (Figure 3d–f) [39,89,108,109,110,111,112]. The methods described above can be used periodically for assessing the prognosis of the patients in relation to iron toxicity, which is directly related to their total body or individual organ iron load and organ function, as well as for the adjustment of the dose protocols during iron chelation therapy (Figure 3) [39,108,109,110,111,112].

In developed countries, the frequency of iron load monitoring and the progress of the chelation therapy in TM and other categories of regularly transfused patients is usually carried out by estimating serum ferritin levels every three months, while liver iron and cardiac iron concentration estimation can be carried out annually by using the MRI T2 and T2* signal intensity relaxation time techniques. In the developing countries where MRI facilities may be absent, serum ferritin levels are mostly used for iron load estimations. It should be noted that in regularly transfused TM and other patients, liver iron appears in most cases to correlate well with serum ferritin levels, but cardiac iron load does not appear to correlate with either liver iron or serum ferritin levels [106,107,108,109].

In most cases, serum ferritin levels greater than 2500 μg/L and transferrin iron saturation of 100% suggest the presence of highly toxic iron levels in the tissues and the need for the use of more intensive chelation therapy protocols and higher chelating drug doses. Despite the overall improvements using the above-established methods of monitoring iron levels, there can be limitations in the prediction of either the total body iron load or the extent of iron toxicity because of many other variables. These may include differences in the total body and organ iron distribution of stored iron, in the iron assessment and detection methods, or in the specificity of iron pool and iron removal effects from various organs using different chelating drugs or chelation therapy protocols [100]. A number of other factors can also influence the iron metabolic processes, or the estimation of the iron load levels as a result of individual variations such as dietary habits, infection, inflammation, rate of iron absorption and excretion, transfusion frequency, erythropoietic activity, bleeding, drug or RBC reactions, etc. [39,113,114,115].

**Figure 4:**
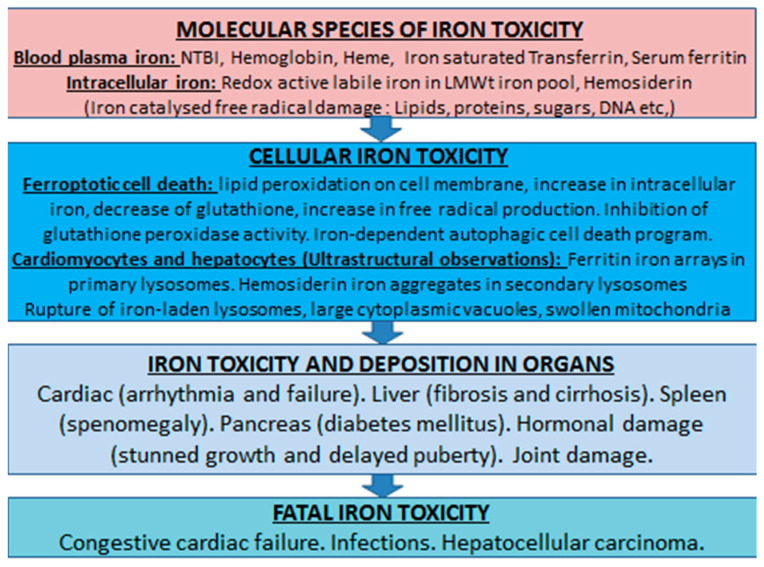
Toxicity aspects of transfusional iron overload from molecular to organ damage. Iron overload toxicity is implicated at different levels and involves progressively molecular, cellular and organ damaging changes, which can lead to fatalities. In each case the impact of toxicity depends on the concentration of excess iron. Congestive cardiac failure is the main cause of mortality in thalassemia major in the absence of effective chelation therapy. Excess iron is also implicated in some fatal cases of infections, liver fibrosis, and hepatocellular carcinoma (NTBI: Non-transferrin bound iron; LMWt: Low molecular weight).

Overall, the factors contributing to iron overload and the diagnostic techniques used for its assessment have played a major role in defining the etiology and the level of iron toxicity in different organs (Figure 4). This information is also critical for monitoring changes in iron load during iron chelation therapy and the design of effective iron chelation therapies for all categories of RBC transfused iron-loaded patients.

## 6. Excess Iron Deposition and Toxicity in Non-Transfusional Iron Loading Conditions

There are many non-transfusion-dependent iron overloading conditions affecting millions of patients worldwide, including idiopathic hemochromatosis, thalassemia intermedia, and transfusion-independent post-allogeneic HCT patients, which was introduced in previous sections (Table 1) [10,42,43,47,116,117,118,119,120,121,122]. In all these and similar conditions, iron overload toxicity affects the function of many organs. In each case, the level and intensity of the toxicity is proportional to the level of iron overload, as shown in transfusional iron loading conditions.

In most cases of non-transfusion-dependent iron overloading conditions, iron toxicity in iron-loaded organs is reversible and the restoration of normal function can be accomplished by the removal of excess iron. The gold standard form of treatment in almost all categories of non-transfusion dependent iron overloading conditions is phlebotomy, whereas chelation therapy is an alternative option for some cases, especially where phlebotomy cannot be used [34,47,116,117,118,119,120,121,122]. The ultimate aim of treatment is similar to that proposed for transfused patients, namely the complete removal of excess iron and the achievement of normal iron store levels. As shown in transfused patients, non-transfusion-dependent iron-loaded patients achieving this stage are devoid of excess iron toxicity and associated complications.

The same standard diagnostic techniques, including MRI T2* and serum ferritin, are used for monitoring iron overload and the progress of therapeutic intervention, including phlebotomy and chelation therapy in idiopathic hemochromatosis, thalassemia intermedia, post-allogeneic HCT patients, and other categories of patients with non-transfusion-dependent iron overloading conditions (Table 1) [122,123,124,125,126,127].

Excess iron deposition in small parts of organs has also been realized in non-iron-loaded patients. The identification of small iron deposits in different parts of the body, which are not linked to iron overloading diseases nor to diseases with abnormal iron metabolism, began in the 1980s and continues until today [128,129,130,131,132,133,134]. In particular, many such findings were related to neurological and neurodegenerative diseases associated with excess iron and detected using earlier versions of MRI signal intensity relaxation time techniques [128,129,130,131,135,136,137,138,139].

The iron toxicity and tissue damage identified in many of the non-iron-loaded categories of patients concerns the presence of focal iron deposits, which are usually detected by MRI T2* and are of major significance in many clinical conditions. In particular, focal iron accumulation in the brain with increased MRI T2* signal intensity has been detected in many neurodegenerative diseases, including Alzheimer’s disease, Parkinson’s disease, Friedreich’s ataxia, and neurodegeneration with brain iron accumulation (NBIA) (Table 1) [140,141,142,143,144]. In the latter, at least fifteen diseases, including pantothenate kinase 2-associated neurodegeneration (PKAN) with NBIA, have been characterized due to iron deposition in different parts of the brain [144].

The re-trafficking of iron is also found in many categories of chronically ill patients with anemia of chronic disease or anemia of inflammation, where the retention of iron in reticuloendothelial cells causes iron-restricted erythropoiesis, low production of hemoglobin, and also anemia. These diseases include cancer and hematological malignancies, infections, inflammatory diseases, immune-mediated diseases, chronic kidney disease, and congestive heart failure (Table 1) [145,146,147,148,149,150,151,152,153]. The treatment of the anemia of chronic disease is very challenging for almost all categories of patients affected and is the subject of research investigations and therapeutic approaches, including, in many cases, the fine balance between iron deficiency level and iron toxicity of administered iron [154,155,156,157,158,159,160,161].

A similar diversion of iron to that observed in the anemia of chronic disease is associated with iron accumulation in the macrophages of cancer patients. In general, macrophages associated with tumors play an important role in both the immune system and tumor microenvironment, and interact with different pathways, including ferroptosis, which are involved in cancer initiation, proliferation, metastasis, in drug resistance, etc. [81,162,163,164,165,166]. In particular, during cancer proliferation, adjacent endothelial and other cells are utilized by cancer cells for energy supply and nutrients, including iron, most of which is released during ferritinophagy. Iron is subsequently effluxed by cancer cells and taken by macrophages, causing the formation of iron-laden macrophages, a reduction in serum iron, and iron deficiency [74,84,85,167,168,169,170]. A concomitant effect of the presence of iron-laden macrophages is the substantial increase in serum ferritin levels called hyperferritinemia, which is a negative prognostic factor for many diseases in addition to cancer, such as different forms of inflammation, autoimmune disorders, and infection [171,172,173,174]. Overall, the immune response in all these conditions appears to lead to activation of macrophages, iron uptake into macrophages, reduction of serum iron, and to increased synthesis and secretion of ferritin in plasma by macrophages [167,168,169,170,171,172,173,174,175].

Excess iron deposition has been identified in many other organs of different categories of patients which are not related to iron overload. These include ovarian endometriosis, multiple sclerosis, cognitive function, depression, liver lesions, and ageing [176,177,178,179,180,181]. The precise mechanisms and the level of contribution of iron toxicity regarding the causes or impact on each of these conditions have not yet been fully clarified.

## 7. Iron Toxicity from Labile Forms of Iron and Other Molecular Interactions

There are thousands of research findings and reports in the medical literature of naturally occurring or xenobiotic molecules, including drugs with metal binding potential, interactions with iron and other metals, and different forms of toxicity [20,182,183,184,185,186]. Each of these molecules and their iron or other metal complexes have diverse biological, pharmacological, and toxicological activity. In cancer, for example, the activity of such molecules may range from pro-oxidant and pro-carcinogenic to antioxidant and anti-carcinogenic effects and also many other effects, including the enhancement or inhibition of ferroptosis [187,188,189,190,191,192,193,194]. Similarly, many old and new carcinogens may contain or interact with iron, including food additives and coloring agents, many industrial and other chemicals, inhaled carcinogens from smoke, and other compounds from environmental pollution (Table 1) [195,196,197]. In particular, iron in heme and the nitroso heme complexes found mainly in processed meat have been implicated in the rise of colorectal and other cancers [198,199,200,201,202,203,204,205,206]. Carcinogenic toxicity is also suspected from other iron complexes present or formed in asbestos fibers, during cigarette smoking, and in barbecuing (Table 1) [207,208,209,210,211,212,213,214].

**Figure 5:**
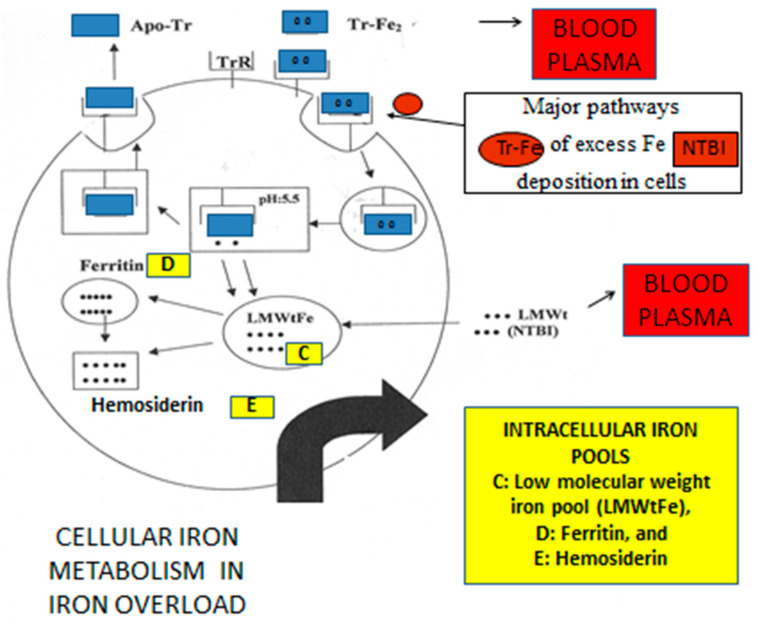
The iron pools and metabolic pathways in iron overload. A cartoon displaying a cell, e.g., hepatocyte, where intracellular iron uptake is mostly accomplished by diferric transferrin (Tr-Fe_2_) through transferrin receptors (TrR), release of iron in a low molecular weight iron pool (LMWtFe) at low pH, and utilization of iron for storage in ferritin and hemosiderin and, to a smaller extent, for the turnover of other iron proteins. Intracellular iron intake in iron overload is also accomplished via low molecular weight (LMWt) non-transferrin bound iron (NTBI). The black dots represent iron molecules.

Changes in iron metabolism with iron toxicity implications are also observed during therapeutic interventions. For example, excess iron release causing increased transferrin saturation and formation of non-transferrin-bound-iron (NTBI) has been observed from tissue damage in iron overloading conditions and during cancer chemotherapy and radiotherapy treatments (Figure 5) [215,216,217,218,219,220,221,222,223,224,225]. Under these conditions, excess iron present in plasma can increase oxidative stress and potentially cause cellular and organ toxicity [226,227]. It can also facilitate microbial growth, mostly affecting immunocompromized cancer, transplanted, and other patients who are more susceptible to infections in comparison to normal individuals [103,228,229]. 

The presence of labile, catalytically active iron forms have been suggested in many other diseases, in addition to those considered that are associated with NTBI (Figure 5). In particular, catalytically active iron has been identified in the urine of different categories of patients, including acute and chronic kidney disease and cardiovascular disease [62,230,231,232].

Other diseases associated with abnormalities in iron metabolism and heterogeneous iron distribution with toxic side effects include those related to heme biosynthesis, mitochondrial iron metabolism, and rare cases of iron overload [21,44,233,234]. Among these are porphyria cutanea tarda, pyruvate kinase deficiency, congenital dyserythropoietic anaemia, congenital atransferrinaemia, etc. (Table 1) [235,236,237,238,239,240]. Many diseases have also been linked or associated to other abnormalities of mitochondrial iron metabolism and of different metabolic changes [241,242,243,244].

The molecular interactions of drugs and dietary molecules with iron and iron metabolic pathways are an area of continuous and thorough investigation regarding iron-related toxicity in the field of general pharmacology, toxicology, and nutrition. Thousands of such interactions of iron with drugs and dietary molecules have been reported in the scientific and medical literature emphasizing the prospect of free radical and other toxicity, especially affecting particular organs and characterized as a toxic side effect of the therapy (Figure 2). For example, anthracyclines, including doxorubicin, can cause acute and chronic cardiotoxicity in cancer patients, with the latter considered responsible for death in about one-third of patients during the long-term use of doxorubicin and long-term cardiotoxicity (Table 1). Labile iron and free radical production have been implicated as one of the mechanisms of anthracycline cardiotoxicity [245,246,247,248]. In this context, the EDTA iron chelator prodrug dexrazoxane has been widely used in cancer patients, including young children, for cardioprotection against doxorubicin and similar drug cardiotoxicity [249,250,251]. In contrast, the kidney is the major target organ of toxicity for cisplatin, an anticancer drug widely used for the treatment of about 50% of patients with different cancer categories [252,253]. In this case, labile iron and hemosiderin iron accumulation were implicated and suspected to play a role in the cisplatin-induced kidney injury [62,254,255,256]. The enhancement of iron-induced free radical toxicity has also been observed by the chelating drug EDTA, with some investigators questioning its safety in alternative medicine (Figure 2) [257,258].

Another area of iron-induced toxicity is related to the mode of siderophore action affecting some drugs with iron binding properties [259,260]. In particular, the iron chelating drug DF has been shown to promote the growth of some bacterial and fungal species with serious infectious toxicity implications, including fatalities [260,261]. In particular, among the established serious toxic side effects of DF are yersiniasis and mucormycosis, which mainly occur in iron-loaded and renal dialysis patients, respectively. In both cases DF appears to act as a siderophore and donates iron for the growth of *Yersinia enterocolitica* and *Zygomyces*, respectively [260,261,262]. The pharmacological and toxicological effects of many other categories of drugs with iron binding potential have not yet been fully investigated [20,259,263,264,265].

In addition to drugs, pharmacological and toxicological implications are expected from hundreds of dietary and biological molecules with iron-binding potential, as well as from their iron complexes (Table 1) [20,184]. For example, the interactions of iron and ascorbic acid (vitamin C), both of which are essential nutrients of nutritional, physiological, pharmacological, and toxicological interest [266]. Millions of people use these two nutrients found in food daily, and as pharmaceutical and nutraceutical preparations. In particular, one of the main functions and use of vitamin C is its antioxidant activity, with a wide range of applications in many diseases and clinical investigations, including cancer and ageing [266,267,268,269,270,271,272,273]. Vitamin C has iron and other metal-binding capacities and is also a reducing agent, forming a complex with iron (III) followed by reduction to iron (II) [266]. The biological and clinical activities of iron, ascorbate, and the ascorbate–iron complex can also be affected by many other nutrients and pharmaceutical preparations, which may reduce or potentiate iron toxicity [274,275,276,277,278]. Similar effects to those shown in the interactions of vitamin C and iron are expected from other biomolecules with iron-chelating potential such as folic acid, lipoic acid, and catecholamines, as well as metal ions competing with iron [59,184,185,279,280,281,282,283].

Abnormalities of iron metabolism with toxicity and other implications regarding the role of regulatory molecules, such as the apical divalent metal transported protein (DMT1), ferroportin, hepcidin, erythroferrone, erythropoietin, and related genomic, redoxomic, transcription, and other factors is currently under evaluation for determining their impact in many diseases and also for the design of new pharmaceuticals [284,285,286,287,288,289,290,291,292]. In particular, the interplay of regulatory molecules and other factors with pharmaceuticals on the regulation of diseases related to iron imbalance and toxicity could facilitate the design of optimal therapies and personalized medicine [293,294].

Despite the complexities and the many factors involved in diseases of iron metabolism and iron toxicity, the identification of the mechanisms involved, and the use of appropriate diagnostic criteria could provide efficient targeted therapies and the complete elimination of iron toxicity, as shown in the case of the transition of thalassaemia from a fatal to a chronic disease [39,88,295,296,297,298].

## 8. Conclusions

The spectrum of diseases associated with abnormalities of iron metabolism linked to iron toxicity and the number of patients affected worldwide is continuously increasing. New research developments, such as the identification of ferroptotic cell death in almost all common and rare diseases and the introduction of new diagnostic techniques such as the MRI T2* for the detection of iron deposits in major organs, have attracted the attention of many investigators working on many diseases and fields of medicine outside the field of iron metabolism and iron toxicity. In particular, the introduction of MRI T2* could not only be used in the monitoring of new chelation and other therapies in transfusional and other iron-loaded categories of patients but also therapies for other organs, such as those associated with the brain of non-iron-loaded categories of patients and many categories of several common neurodegenerative diseases, including Alzheimer’s disease and Parkinson’s disease. Furthermore, progress in the molecular interactions of iron and the prospect of increasing toxicity involving dietary, biochemical, xenobiotic molecules, and drugs have also attracted the interest of different specializations in medicine and the general public. In particular, the association of processed meat implicating heme and nitroso heme complexes in carcinogenesis and also mesothelioma caused by asbestos fibers containing iron, raised the alarm in relation to different forms of iron toxicity from dietary and environmental factors. Similarly, research interest is also increasing in relation to the implication of iron interactions in the toxic side effects of widely-used drugs such a doxorubicin and cisplatin or the decrease of efficacy in other widely-used drugs such as tetracyclines and hydroxyurea. Many other developments regarding the molecular and cellular effects of iron toxicity have also been identified and reported in a number of other fields of iron metabolism and diseases such as cancer and the anemia of chronic disease, which affects millions of patients.

Despite the improvements in identifying the mechanisms and in the methods of diagnosis of iron toxicity, the complexities and the involvement of many factors associated with different forms of iron toxicity have not yet led to improvements in many therapeutic fields for many diseases. However, there are exceptions, and progress in the therapeutic outcomes has been reported for some diseases, especially in the treatment of transfusional iron overload in TM, the disease with the highest rate of mortality and morbidity related to iron toxicity worldwide. In particular, the identification of the mechanisms of iron toxicity involved and the use of appropriate diagnostic criteria, including MRI T2*, helped in the introduction of effective targeted chelation therapies, which has overall resulted in the complete elimination of excess iron from the body of TM patients and also of iron toxicity, eventually causing the transition of thalassaemia from a fatal to a chronic disease.

## Figures and Tables

**Table 1:** Iron overload and other categories of iron toxicity.

A. Transfusional and other iron-loaded categories of patients
(a) Thalassemia
β-thalassemia major
β-thalassaemia intermedia: Heterozygous mild β+/severe β+. Homozygous β (o) or β+ with other genetic factors leading to increased γ chain production. Homozygous normal Hb A2 β thalassemia (type I). Heterozygous β (o) or β+/Normal Hb A2 β thalassemia (type I).
δβ thalasemia and High Persistent Fetal Hemoglobin (HPFH): Homozygous (G) γ, δβ thalasemia. Heterozygous β (o) or β+ thalasemia/δβ thalasemia. Heterozygous Greek HPFH/β thalassemia.
β or δβ thalasemia with structural hemoglobin variants: Hemoglobin S,C,E in association with β (o), β+ or δβ thalasemia. Hb E thalassemia. Hb E/β thalassemia
Severe heterozygous β thalasemia
Interactions of α and β thalasemia: α- and β-thalassemia
Interactions of α thalasemia and Hemoglobin S
(b) Sickle cell anemia
(c) Hematologic categories: Myelodysplastic syndrome, aplastic anemia, Fanconi anemia, Blackfan-Diamond anemia, pyruvate kinase deficiency, hematopoietic stem cell transplantation, hereditary sideroblastic anemia.
(d) Oncologic and other categories: Cancer, renal dialysis
**B. Idiopathic hemochromatosis and other diseases related to increased iron absorption**
Iron overload in liver disease. Iron poisoning. Dietary or iatrogenic iron intake.
**C. Hemolytic and other anemias:** Hemolytic diseases. Pernicious anemia. Prophyria cutanea tarda. Hereditary spherocytosis. Congenital dyserythropoietic anemia. Congenital atransferrinemia. Hereditary hypochromic anemia.
**D. Anemia of chronic disease: Inflammatory diseases, chronic kidney disease, cancer etc.**
**E. Neurodegenerative diseases:** Friedreich’s ataxia, Alzheimer’s disease, Parkinson’s disease, etc.
**F. Drug and natural product iron toxicity:** Drug toxicity, e.g., doxorubicin. Natural product toxicity, e.g., ascorbic acid
**G. Dietary toxins:** Processed meat carcinogens. Red meat carcinogens. Nitroso heme complexes. Other heme and iron complexes.
**H. Environmental toxins:** Toxic iron particles from gas fumes. Asbestos.
**I. Free radical toxicity:** Diseases of free radical pathology. Ischemia reperfusion injury. Storage of organs for transplantation.
**K. Ferroptotic cell death related diseases:** Almost all common and rare diseases.
**L. Iron toxicity associated with abnormalities related to regulatory proteins of iron metabolism**

## Data Availability

Not applicable.

## Abbreviations

DF	deferoxamine
DFRA	deferasirox
DMT1	divalent metal transported protein
EDTA	ethylenediaminetetraacetic acid
GPX4	glutathione peroxidase
HCT	hematopoietic stem cell transplantation
Hb	hemoglobin
HPFH	high persistent fetal hemoglobin
L1	deferiprone
LMWt	low molecular weight
MRI	magnetic resonance imaging
NBIA	neurodegeneration with brain iron accumulation
NTBI	non-transferrin bound iron
PKAN	pantothenate kinase 2-associated neurodegeneration
RBC	red blood cell
SCD	sickle cell disease
TI	thalassemia intermedia
TM	beta thalassemia major
